# Informing Evidence-Based Decision-Making for Patients with Comorbidity: Availability of Necessary Information in Clinical Trials for Chronic Diseases

**DOI:** 10.1371/journal.pone.0041601

**Published:** 2012-08-03

**Authors:** Cynthia M. Boyd, Daniela Vollenweider, Milo A. Puhan

**Affiliations:** 1 Division of Geriatric Medicine and Gerontology, Department of Medicine, Johns Hopkins University, Baltimore, Maryland, United States of America; 2 Division of General Internal Medicine, Department of Medicine, Johns Hopkins University, Baltimore, Maryland, United States of America; 3 Department of Epidemiology, Johns Hopkins Bloomberg School of Public Health, Maryland, United States of America; University Paris Descartes, France

## Abstract

**Background:**

The population with multiple chronic conditions is growing. Prior studies indicate that patients with comorbidities are frequently excluded from trials but do not address whether information is available in trials to draw conclusions about treatment effects for these patients.

**Methods and Findings:**

We conducted a literature survey of trials from 11 Cochrane Reviews for four chronic diseases (diabetes, heart failure, chronic obstructive pulmonary disease, and stroke). The Cochrane Reviews systematically identified and summarized trials on the effectiveness of diuretics, metformin, anticoagulants, longacting beta-agonists alone or in combination with inhaled corticosteroids, lipid lowering agents, exercise and diet. Eligible studies were reports of trials included in the Cochrane reviews and additional papers that described the methods of these trials. We assessed the exclusion and inclusion of people with comorbidities, the reporting of comorbidities, and whether comorbidities were considered as potential modifiers of treatment effects. Overall, the replicability of both the inclusion criteria (mean [standard deviation (SD)]: 6.0 (2.1), range (min-max): 1–9.5) and exclusion criteria(mean(SD): 5.3 (2.1), range: 1–9.5) was only moderate. Trials excluded patients with many common comorbidities. The proportion of exclusions for comorbidities ranged from 0–42 percent for heart failure, 0–55 percent for COPD, 0–44 percent for diabetes, and 0–39 percent for stroke. Seventy of the 161 trials (43.5%) described the prevalence of any comorbidity among participants with the index disease. The reporting of comorbidities in trials was very limited, in terms of reporting an operational definition and method of ascertainment for the presence of comorbidity and treatments for the comorbidity. It was even less common that the trials assessed whether comorbidities were potential modifiers of treatment effects.

**Conclusions:**

Comorbidities receive little attention in chronic disease trials. Given the public health importance of people with multiple chronic conditions, trials should better report on comorbidities and assess the effect comorbidities have on treatment outcomes.

## Introduction

Recent reports have posed concerns about the challenges of caring for a growing population with multiple chronic conditions [1]–[7]. This population faces self-management challenges and adverse events and often experiences high health care utilization and poor quality of care [8], [9]. One concern is an inadequate evidence base. The roots of this problem extend throughout the translational path, from the generation of the evidence to the synthesis of the evidence upon which clinical practice guidelines depend [1]. Comorbid patients are frequently excluded from the relevant clinical trials [10]–[12]. And even when such patients are not explicitly excluded, it is often difficult to know whether the overall effect estimate can be extrapolated to individuals with comorbidities, or whether the effect of a treatment is different in those with or without comorbidities. Also the applicability of “average” trial results to specific individuals, or particular groups, such as complex patients with more competing risks than average, remains challenging [13]–[16]. These uncertainties about treatment effects in comorbid patients translate into systematic reviews and guideline development, making it difficult to establish treatment recommendations for these patients [1].

We designed this project to address these methodological issues. Many trials focus on treatments for a single condition, and may exclude people with comorbidities, and the typical primary hypothesis does not include effect modification. With rare exceptions, systematic reviews and guidelines also emphasize the single-disease perspective as they inherit the limitations of the trials and studies they review. For systematic reviews, the scientific community has not identified the best methods of determining and describing applicability of evidence for comorbid patients [17]. Current methods for systematic reviews do not include explicit criteria as to how to frame a question and search for and evaluate evidence for a specific comorbid population. For example, should the scientific community value a study with a lower quality of evidence, in terms of rigor of design, because it has a greater inclusion and analytic attention on comorbidity, or is a more rigorous design preferred, even if the study is less focused on comorbidity or reports less of the necessary information. It is also unknown how many existing studies contain the necessary information to address comorbid patients in systematic reviews, and therefore in for clinical decision-making.

Diabetes, congestive heart failure, chronic obstructive pulmonary disease (COPD), and stroke may serve as typical conditions where we commonly see these methodological issues. Both older and younger people with these conditions often suffer from comorbidities [18]–[20]. While the number varies depending on the demographics of the population and the number of comorbidities assessed, less than 20% of people with any these conditions have the condition in isolation [18]–[20]. It is unclear whether the reporting of comorbidities in trials focused on these conditions allows for the extraction of information in order to consider comorbidity in systematic reviews. If reporting is insufficient, clinicians, guideline developers, and policy makers will not be able to make well-informed recommendations for clinical practice. Therefore, the aim of this study was to assess the inclusion of people with comorbidities and the reporting of comorbidities in trials of these four major chronic diseases, and whether comorbidities were considered as potential modifiers of treatment effects.

## Methods

### Study Design and Selection

We conducted a survey of trials reporting on drug and non-drug interventions in patients with the four common chronic diseases: COPD, heart failure, stroke and type II diabetes mellitus. Some results of this survey, addressing a different question, have been published earlier [21].

Ethics Statement: This work uses data in the published literature, and thus institutional review board approval was not sought.

We chose diseases associated with high morbidity, mortality and significant health care expenditures [22]–[24]. We focused on widely prescribed drug and non-drug therapies, and we wanted to have a complete set of trials. Therefore, we based the selection of randomized controlled trials (RCTs) on 11 Cochrane Reviews that systematically identified and summarized RCTs from 1944–2009 on the effectiveness of diuretics, metformin, anticoagulants, long-acting beta agonists alone or in combination with inhaled corticosteroids, lipid lowering agents, and the non-drug interventions exercise and diet for each of the four diseases [25]–[35]. The Cochrane reviews described the search strategy and eligibility criteria. We retrieved the main reports of included RCTs and retrieved additional papers that described the methods of these trials. We did not consider abstracts and unpublished data used in the Cochrane reviews because they could not provide the level of detail that we needed. We excluded 22 trials (out of 183) for this reason. The bibliography of excluded trials is available on request.

### Data Extraction

Before systematically extracting data from each trial, we developed a codebook that provided a detailed description of the information to be extracted and how to score it. We pilot tested the data extraction forms and the codebook on a random sample of 10 articles. One reviewer extracted all data and at least one other reviewer checked the data. They recorded and discussed all disagreements. If they were not able to resolve an issue, a third reviewer examined the article and all three reviewers discussed the issue until they resolved it.

### Items related to Inclusion and Exclusion Criteria

#### Reporting and Replicability of Inclusion and Exclusion Criteria

In order for us to better understand the population for whom researchers assessed treatment effects, the trial must clearly define inclusion and exclusion criteria. This is one criterion used for gauging how to generalize treatment effect estimates to a specific patient or group of patients [10], [13], [36], [37]. We recorded whether or not each trial reported its inclusion and exclusion criteria. Of those studies that had inclusion and exclusion criteria, using a 10-point Likert-type scale [1 =  not replicable, 10 =  perfectly replicable, 5 =  moderate (e.g. replicable for some criteria, irreplicable for others)], two investigators independently rated the replicability of the inclusion and exclusion criteria. More specifically, they rated the difficulty of applying the inclusion and exclusion criteria, as stated in the trial reports, to specific patients. We assessed the inter-rater agreement using the concordance correlation coefficient rho. To aggregate the two independent ratings, we took the mean of the two ratings for trials where the disagreement was <3 points. If disagreements in ratings were ≥3 points for any given trial, we rerated replicability and resolved disagreements through discussion between the two investigators.

#### Specific Exclusion Criteria

We recorded whether or not the trials excluded people with the following conditions: pregnancy, recent or acute respiratory tract infections (pneumonia or COPD exacerbations), asthma or atopic conditions, COPD or emphysema, other lung disease (not asthma or COPD), coronary artery disease (history of myocardial infarction or angina), hypertension, arrhythmias, unspecified cardiac conditions (e.g. cardiovascular disease unspecified), renal insufficiency, liver insufficiency, heart failure, New York Heart Association Class IV Heart Failure, valvular disease, type II diabetes mellitus, type I diabetes mellitus, insulin therapy, damage from diabetes mellitus (nephropathy, retinopathy, neuropathy), peripheral vascular disease, oxygen therapy, oral steroid use, unspecified cancer, lung cancer, anemia, musculoskeletal diseases or disabilities, neurologic disabilities, other brain injuries (including hemorrhagic stroke), unable to exercise (unspecified), psychiatric illness, impaired mental status, and serious concomitant disease (unspecified). We chose conditions which are believed to be prevalent or potentially represent important treatment effect modifiers for these conditions [19], [37], [38]. We also recorded whether there were specific age exclusions. Disagreements in the categorization of these exclusion criteria were resolved by discussion. If a “severe” version of one of these conditions was an exclusion criterion, we recorded it as an exclusion, even if a mild form of the condition was not an exclusion criterion for the trial. We also recorded if the trial had exclusion criteria other than these listed above or not.

### Reporting of Definitions for the Presence or Absence of Comorbidities, Ascertainment Method and Use of Treatments

We were also interested in how often comorbidities were included as part of the description of the characteristics of the trial participants as selection procedures and trial participation also affect the characteristics of the trial participants. We recorded whether or not the study described a definition for those comorbidities for which prevalence was reported, and how the study obtained the information needed for this definition [(patient reported, medical record (includes doctor-reported), not reported, confirmed at baseline (like echo, blood tests, etc), others (e.g. questionnaires)]. We also recorded whether the studies reported any treatments for the comorbidities for which prevalence was reported. The comorbidities assessed were osteoporosis, diabetes mellitus, COPD, hypertension, coronary artery disease (angina or myocardial infarction), sleep apnea, heart failure, cancer, gastro-esophageal reflux, depression, dyslipidemia, osteoarthritis or joint pain, urinary problems, gout, chronic back pain or spinal stenosis, stroke, liver disease, chronic kidney disease, lung disease other than COPD or asthma, peripheral vascular disease, anxiety, arrhythmias, dementia or cognitive impairment, and obesity.

### Are Comorbidities Treatment Effect Modifiers? (Subgroup Analyses)

Recognizing that authors have limited power to pre-plan subgroup analyses, we assessed how often and the way the study performed subgroup analyses using recently described criteria [21], [39], [40]. We defined a subgroup analysis as the analysis of an effect that changed (or did not change) according to different levels of a variable measured before randomization. For those trials that did report one or more subgroup effects, we recorded whether the subgroup was related to a comorbidity. For those trials that reported a subgroup based on a comorbidity, we recorded whether these trials used an interaction term to compare effects across subgroups by comorbidity status and whether the trials assessed if the interaction terms were statistically independent from other subgroup effects.

### Competing Risks and Heterogeneity of Treatment Effect Due to Different Baseline Risks

We recorded whether the trials considering competing risks in the design and analysis of trials. Competing risks are defined as events that occur when the time to a disease-specific endpoint of interest may be precluded by death or a major health event from another cause, and are increasingly recognized as relevant to decision making for older adults and people with multiple chronic conditions [16], [41]–[44]. We also were interested in whether or not the trials reported on the heterogeneity of treatments effects due to different baseline risks.

### Statistical Analysis

We used descriptive statistics to summarize our findings across the entire set of trials, and stratified by disease and type of treatment (drug vs. non-drug). We conducted all analyses using STATA for Windows version 10.1 (Stata Corp., College Station, Texas, U.S.).

## Results

We included 161 trials. 91 percent (n = 147) reported inclusion criteria and 85 percent (n = 137) reported exclusion criteria. The replicability of inclusion and exclusion criteria was moderate in general. (See Table 1) Overall, for all four chronic diseases, the replicability of inclusion criteria was mean [standard deviation (SD)]: 6.0 (2.1), range (min-max): 1–9.5. The inter-rater agreement was good, with a concordance correlation coefficient rho of 0.82 [(95% confidence interval (CI) 0.77–0.87)] and a mean difference (SD) between reviewers of 0.13 (1.35). For all four chronic diseases, the replicability of exclusion criteria was slightly worse, with a mean (SD) of 5.3 (2.1), range (min-max): 1–9.5. Again, the inter-rater agreement was good, with a concordance correlation coefficient rho of 0.81 (95% CI 0.76–0.87), and a mean difference (SD) between reviewers of 0.02 (1.38). Replicability did not vary much across diseases. (Table 1).

**Table 1:** Replicability of Inclusion and Exclusion criteria, by disease.

		Mean(SD)	Range (min-max)
Heart Failure	Inclusion criteria	5.3 (2.3)	1–8.5
	Exclusion criteria	5.5 (2.5)	1–9.5
COPD	Inclusion criteria	6.5 (1.8)	1.5–9.5
	Exclusion criteria	5.5 (1.6)	1–8.5
Diabetes	Inclusion criteria	5.5 (2.2)	1–9.5
	Exclusion criteria	4.5 (2.5)	1–9
Stroke	Inclusion criteria	6.8 (1.9)	2–9.5
	Exclusion criteria	5.9 (2.0)	1–9

**Figure 1:**
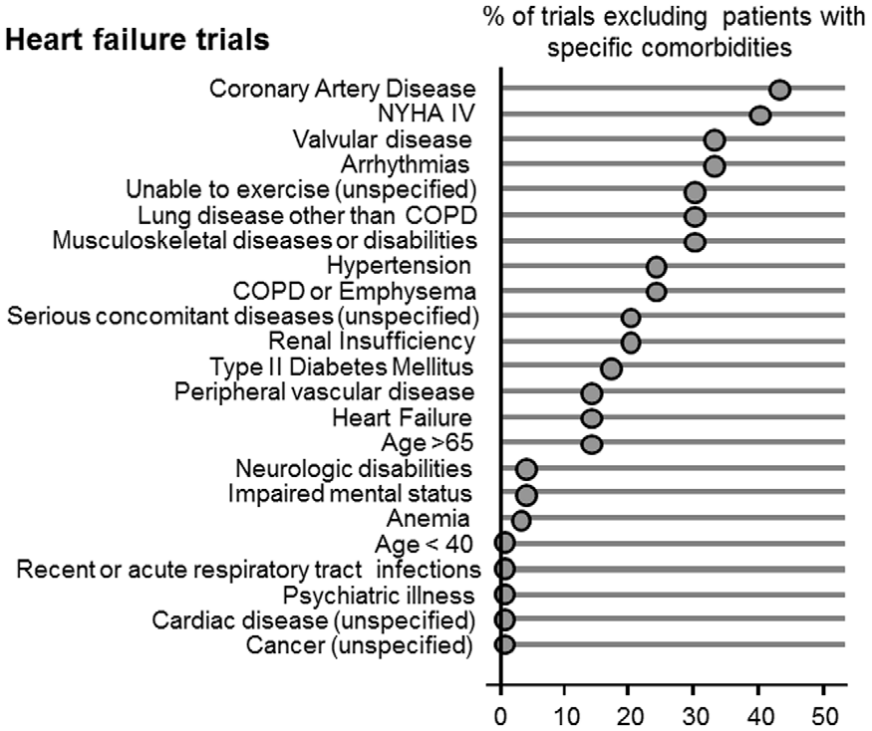
Proportion of heart failure trials where patients with specific comorbidities were excluded.

**Figure 2:**
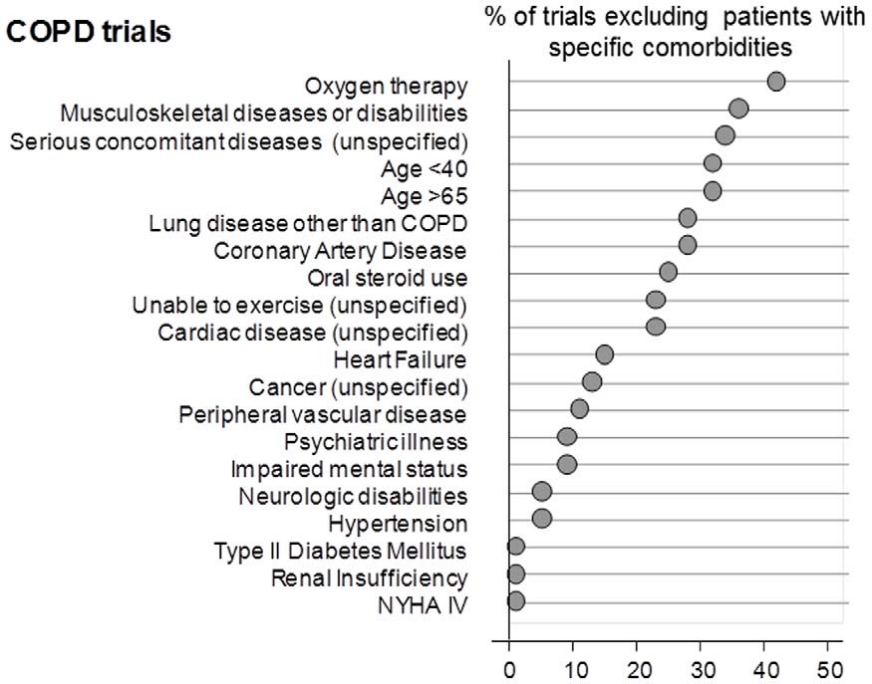
Proportion of chronic obstructive pulmonary disease (COPD) trials where patients with specific comorbidities were excluded.

**Figure 3:**
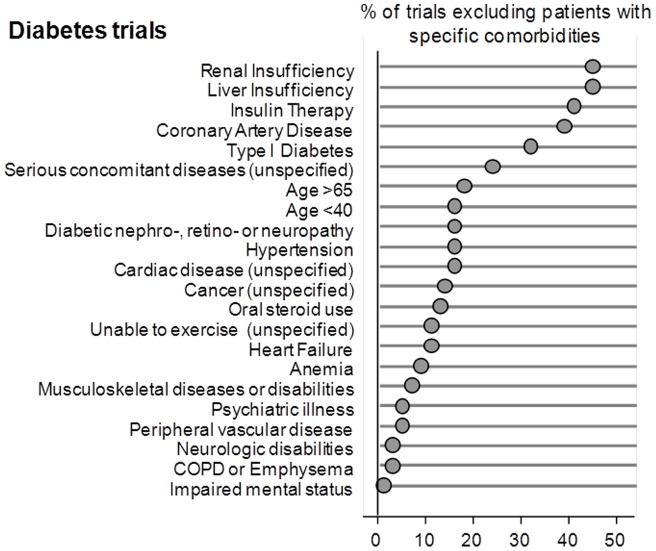
Proportion of diabetes trials where patients with specific comorbidities were excluded.

**Figure 4:**
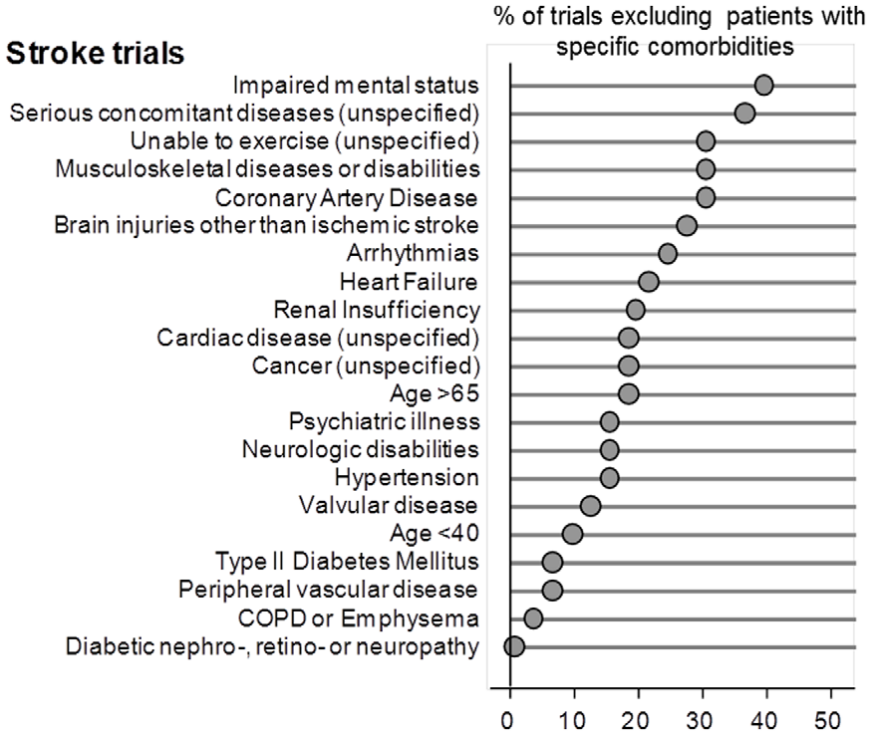
Proportion of stroke trials where patients with specific comorbidities were excluded.

The proportion of exclusions for comorbidities ranged from 0 to 42 percent for heart failure trials, 0 to 55 percent for COPD trials, 0 to 44 percent for diabetes trials, and 0 to 39 percent for stroke trials. (see Figures 1,2,3, and 4). Twenty-one percent of all trials had an older age exclusion of either age greater than 65, 75 or 80 years of age (13 percent for heart failure trials, 31 percent for COPD trials, 17 percent for diabetes trials, and 18 percent for stroke trials). Serious concomitant illness was a frequent exclusion, with 19 percent of heart failure trials having this exclusion, 33 percent of COPD trials, 23 percent of diabetes trials, and 36 percent of stroke trials) Considering the prevalence of comorbidities among patients with these four index conditions, people with common comorbidities were frequently excluded. For example, 42 percent of heart failure trials excluded people with coronary artery disease, 19 percent of heart failure trials excluded people with renal insufficiency. Similarly, 38 percent of diabetes trials excluded people with coronary artery disease and 44% excluded people with renal insufficiency. Twenty-seven percent of COPD trials excluded people with coronary artery disease, and 35 percent excluded people with musculoskeletal diseases. Among stroke trials, 30% excluded people with coronary artery disease and 24% excluded people with heart failure. The prevalence of the specific exclusions is reported in the Figure.

Seventy of the 161 trials (43.5%) described the prevalence of comorbidities among participants with the index disease. The median number (interquartile range) of comorbidities reported in those 70 trials was 3 (2–4), with 237 total comorbidities reported in these 70 trials. The majority of the 70 trials did not report definitions for these 237 comorbidities; only forty-five (19.0%) of the reported 237 comorbidities in the 70 trials had a definition of the comorbidity. These trials ascertained comorbidity from patient report for 19 (8.0%) comorbidities, from baseline examination for 73 (30.8%), from medical records for seven (3.0%) and from other methods for five (2.1%). The trials did not report the data source they used to ascertain the presence or absence of comorbidities for 133 (56.1%) out of the 237 comorbidities. The studies infrequently described whether or not a person received treatment for a comorbidity. The studies described treatments for 40 (16.9%) of the 237 comorbidities, whereas the studies did not describe treatments for 193 (81.3%) comorbidities and were unclear for 4 (0.2%) comorbidities.

Very few trials used the presence of a comorbidity as a subgroup variable to examine whether it is an effect modifier (n = 5/161, 3.1%). Importantly, none of them used interaction terms to formally test for effect modification. Only one trial (0.6%) considered heterogeneity of treatment effect due to different baseline risks. No trials considered the effects of competing risks [16].

## Discussion

Our analysis, based on studies of patients with any of four major diseases, found that evidence on the effect of treatments on comorbid patients is limited. The replicability of the inclusion and exclusion criteria is only moderate, thus making it hard to judge whether a specific patient or patient population would have been eligible to participate, and making it hard to replicate the clinical trial. Trials excluded patients with many common comorbidities. The reporting of comorbidities in trials of these four major chronic diseases was very limited, and it was even less common that the studies assessed whether comorbidities were potential modifiers of treatment effects.

Prior work has posited that comorbidities and older age are frequent exclusions for clinical trials [10]–[12]. Published reports of trials frequently modify eligibility criteria when compared to the original trial protocols, and that many of these relate to comorbidity [45]. Our results build on this prior work that identifies a potential problem in the literature, in general, by examining the specific information needed to inform clinical decision-making in people with comorbidities, and highlight the challenges of using the current evidence base to determine how we should treat the rapidly increasing population of people with multiple chronic conditions [1]–[3]. The replicability of inclusion and exclusion criteria was poor in many of these studies, and only moderate on average, making it difficult to judge whether a patient or a specific patient population would have been eligible for the trial. This also makes it more difficult to replicate the study and compare populations across studies. Comparing populations across studies is an essential step for systematic reviews that attempt to synthesize the evidence base for specific clinical questions.

Serious concomitant diseases were very common exclusion criteria for trials, and yet how to replicate these determinations was not clear. The existence of multiple common comorbidities was a reason for exclusion in studies examining each of the four chronic diseases. For example, while 55 percent of COPD patients in a population-based study, the National Health and Nutrition Examination Study (NHANES) have arthritis, 35 percent of the COPD trials excluded people with musculoskeletal diseases [38]. Sixteen percent of 45–64 year old diabetic patients, and thirty percent of 65 and older diabetic patients, have low glomerular filtration rate [37]. However, 44 percent of the diabetes trials excluded patients with renal insufficiency. More than 60% of the congestive heart failure that occurs in the United States general population might be attributable to coronary heart disease, yet more than 40% of heart failure trials excluded people with coronary heart disease [46]. The mismatch of eligibility criteria and the characteristics of a patient or patient population with the disease, affects our confidence in applying the results of the trial to these patients or patient populations. However, it is important to note that inclusion/exclusion criteria is an incomplete approach for determining whether a trial's results apply to a patient or a patient population with comorbidities, because restricting a trial population does not necessarily mean that the results do not apply to those excluded from the trial.

Also important is understanding what the patient characteristics were of people enrolled in a trial [13]. Inclusion and exclusion criteria capture who was eligible, but may not reflect who was actually recruited for the trial. For example, even if older adults are not excluded from a trial, if the mean age is 50 years, with a SD of 15, these results may not apply to an 80-year-old man with multiple chronic conditions. Less than half of the trials reported the prevalence of comorbidities. Among those studies that reported prevalence of any comorbidities, the average number of comorbidities reported on was three. The trials also infrequently reported the definition of comorbidity and how the information needed for these definitions was obtained. Thus, determining the comorbidity burden on average of people in these trials was next to impossible. And even if the trials reported this information, we need additional information to determine whether the results of the trials really apply to people with a specific comorbidity (or comorbidities) or risk profile. Heterogeneity of treatment effects may arise from differences in baseline risk of the primary outcome, risk of harm, competing risks, or relative risk reduction [16], [43], [47], [48]. Frequently, researchers examine subgroup effects to determine whether treatment effects vary across groups, but such analyses should be consistent with criteria for appropriate subgroup analyses [39]. While the trials occasionally examined subgroups based on comorbidity status, they were never examined according to established criteria [39]. The trials also rarely considered heterogeneity of baseline risk and competing risks. The result is that it is rare that we can draw conclusions about the presence, or absence, of different treatment effects in people with comorbidity.

While our results may paint a grim picture of the current ability to draw evidence-based conclusions in evidence syntheses about people with comorbidity, they highlight specific steps for improving the way clinical trials, systematic reviews and resulting clinical practice guidelines might better inform treatments of patients with comorbidities. We could overcome some of the limitations of the current evidence base by improved reporting of the eligibility criteria of trials and the comorbidities of patients included in trials. Greater specificity to the descriptions of inclusion and exclusion criteria would help maximize replicability. Tables of baseline characteristics could include more detailed information about single comorbidities, how frequently they occur in combination and their treatments. And although it is often challenging to assess how treatment effects differ according the extent of comorbidity (sample size requirements may exceed feasibility), trials need to address questions of effect modification for common and important comorbidities for which there are prior hypotheses about potential effect modification. One potential solution could be to use observational studies to investigate effect modification by comorbidity [49]. Although observational studies are more prone to confounding, the evidence base could still be improved overall. Defining multimorbidity clearly in such analyses of trials and observational studies will be essential [50]. Another question is how to best convey the uncertainty arising from an incomplete evidence base to patients and health care providers. One solution would be to develop explicit guidance on how to rate the quality of evidence that is used for comorbid patients, which could then inform the strength of recommendations.

Limitations of our study included our focus on trials included in systematic reviews for four chronic diseases. Information about trials in additional chronic diseases may better guide treatment for people with comorbidity [51], [52]. Nevertheless, we chose Cochrane systematic reviews as the basis of our included trials in order to address the important challenge of how evidence syntheses can better address the important challenge of developing evidence-based guidance for people with comorbidity. We chose this system for feasibility, and also to ensure replicable sampling of trials included in our study. While the Cochrane reviews are recent, some of the included studies are significantly older, and there may be differences over time in the extent comorbidities are considered and there were no exclusions in the Cochrane reviews for quality of evidence or risk of bias. We did not assess for changes in reporting over time. However, recent work on exclusion and inclusion criteria suggests that there is not variation over the last 15 years among a more selected group of studies from high-impact journals [11]. Our approach of classifying the exclusions was based on categories of exclusion, and may have grouped exclusions with varied definitions. For example, across trials, there was not a standard definition or threshold of “renal insufficiency”, and so some trials may have excluded only more severe renal disease, whereas some may have had a more restrictive cutpoint. Often, these operational details of a definition are not reported by the trials, as shown in our results.

## Conclusion

Comorbidities receive little attention in chronic disease trials, which is illustrated by exclusion of patients with comorbidities, poor reporting on the prevalence of comorbidities and description of how comorbidities are ascertained, and few investigations into how comorbidities influence the effects of treatments. With the increasing prevalence and public health importance of comorbid patients, [53] clinical trials should not only focus on an index disease but should report on comorbidities, and better assess the effect comorbidities have on treatment outcomes. In this way, systematic reviews based on these trials and resulting clinical practice guidelines would be more relevant for comorbid patients, who contribute substantially to the overall disease burden of populations.

The Corresponding Author(CB) has the right to grant on behalf of all authors and does grant on behalf of all authors, an exclusive license (or non exclusive for government employees) on a worldwide basis to PLOS One.

No patient data were collected. The data presented here are available on request from the authors (Dr. Cynthia Boyd cyboyd@jhmi.edu).
